# Properties of Rubberized Engineered Cementitious Composites Containing Nano-Silica

**DOI:** 10.3390/ma14133765

**Published:** 2021-07-05

**Authors:** Rubendran Loganathan, Bashar S. Mohammed

**Affiliations:** Department of Civil and Environmental Engineering, Universiti Teknologi PETRONAS (UTP), Bandar Seri Iskandar 32610, Perak, Malaysia; rubendran_18000157@utp.edu.my

**Keywords:** engineered cementitious composite, crumb rubber, response surface methodology, elastic modulus, drying shrinkage, nano-silica

## Abstract

To avoid explosive spalling during elevated temperature, crumb rubber (CR) is being added to the manufacturing of engineered cementitious composites (ECC). However, the addition of CR particles adversely affects the mechanical properties of ECC. Therefore, to overcome this issue, nano-silica (NS) is added into rubberized ECC mixture as cementitious material additives. Response surface methodology (RSM) has been utilized to optimize the mixtures of the rubberized ECC with variables: CR (0, 2.5, and 5 vol.%), polyvinyl alcohol (PVA) fiber (0, 1, and 2 vol.%), NS (0, 1, and 2 vol.%), and fly ash (0, 25, and 50 vol.%). The experimentally measured responses are flexural strength, direct tensile strength, elastic modulus, Poisson’s ratio, creep, and drying shrinkage. Mathematical models to predict the targeted responses have been developed using RSM. As a result, a high correlation between the factors and responses has been exhibited by the developed models and the accuracy of fit, where less than 9.38% of the variation was found between the predicted and validated results. The experimental results revealed that the rubberized ECC with the incorporation of nano-silica exhibited a higher compressive strength, direct tensile strength, flexural strength, elastic modulus, Poisson’s ratio, and lower drying shrinkage.

## 1. Introduction

Scrap tires are one of the world’s largest solid wastes and more than 500 million units of waste tires are deposited each year before any type of treatment [1]. In Malaysia, the amount of annually produced scrap tires is in the range of 8.2 million tons, and about 60% of the scrap tires are deposited through unknown channels [2,3]. Most of these scrap tires are generally substantial, bulky and occupy unnecessary space in landfills. The accumulation of scrap tires in abandoned places is a perfect area for a breeding floor for mosquitoes and pests, which, in turn, can be deadly to humans. Additionally, scrap tires are also known to be a non-biodegradable item [4,5]. One solution to counteract this environmental problem is by incorporating crumb rubber (CR) from scrap tires into the production of construction and building materials, such as concrete [6]. CR is defined as the end product of rubber that has been recycled from vehicle tires [7].

Mohammed et al. [8] confirmed that the utilization of CR in concrete as a partial replacement to fine aggregate by volume has led to improve the properties of concrete, which has been named “rubbercrete”. As compared to normal concrete, rubbercrete shows better acoustic properties [9], lower thermal conductivity [10], higher electrical resistivity [11], and more ductility [12], as well as a lower self-weight (own weight of body, due to the mass present in it) [13]. However, as the percentage of CR increases, the strengths (compressive, tensile, and flexural) and elastic modulus of rubbercrete decreases [14,15]. This is attributed to the hydrophobic nature of the CR particles, which repel water and entraps air on their surface [10,11,15,16]. This is the main reason why the interfacial transition zone (ITZ) between CR particles and the hardened cement matrix becomes thicker and weaker [10], which consequently leads to a reduction in the strength of rubbercrete [14]. Thus, researchers have attempted to resolve the problem of the reduction in strengths of rubbercrete using several methods. The widely used methods to restore the lost strength is either through surface treatment of the crumb rubber before mixing with concrete or by the inclusion of cementitious additives into the concrete [17]. One of the additives is nano-silica (NS), which has been utilized in the production of rubbercrete, and its primary purpose is to enhance and restore the strength of rubbercrete through dual physico-chemical functions. Through the chemical process, nano-silica reacts with calcium hydroxide, which is discharged by the hydration process from the cement and subsequently cultivates the production of calcium silicate hydrate (C–S–H) gel which restores strength. In the physical process, nano-silica also acts as a nanofiller. Both functions of NS lead to densifying the microstructure of the hardened cement matrix and the ITZ [8].

On the other hand, engineered cementitious composite (ECC) is a distinctive type of high-performance, fiber-reinforced cementitious composite (HPFRCC) in accordance with the micromechanics principle and the mechanics of fracture [18]. ECC possesses multiple cracks whereas the width openings of the cracks are generally less than 100 μm [19]. As is well documented in the literature, ECC exhibits tensile strength within the range of 4–6 MPa, a compression strain of 0.4–0.65% [3], and compressive strength in the range of 30–80 MPa [20].

Despite the advantages of ECC, its main drawback is explosive spalling when it is being subjected to rapid elevated temperature during fires [21,22,23]. Under a fire situation, the entrapped water and chemically bound water evaporate. Due to the highly densified and disconnected pore system of the ECC, the internal vapor is entrapped in the ECC and will not find its way out [24]. This leads to a high internal pressure build up, followed by explosive spalling [25]. To ease this problem, researchers have incorporated CR in the ECC mixture to avoid explosive spalling in case of a fire. As the CR particles in the ECC would melt under the heat, the residue would connect the pores and consequently provide a passage for vapor to escape. However, by adding crumb rubber to ECC, it could facilitate this situation and explosive spalling would not occur; however, at the same time, to maintain the strength of the ECC, nano-silica has been included to overcome the reduction in ECC strength as well as of the modulus elasticity (ME).

The inclusion of CR into concrete had attracted plenty of attention in the construction industry with regards to how building materials are prepared. As previously mentioned, the innovative aspects of this study, which include CR particles into ECC, could solve a fundamental problem pertaining to when samples encounter fire. This solution would, not only be beneficial to the way building materials are utilized, but also further research could be done on a larger scale.

## 2. Experimental Program

### 2.1. Material Properties

**Figure 1:**
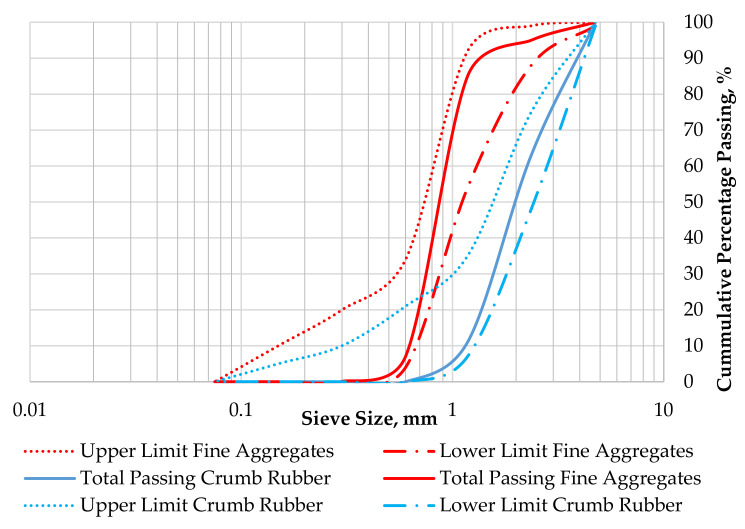
Gradation curves for fine aggregates and crumb rubber.

The cementitious material used in preparation of the ECC mixtures were ordinary Portland cement (OPC, Tasek Corporation Berhad, Ipoh, Perak, Malaysia), fly ash (FA, YTL Cement Berhad, Kuala Lumpur, Malaysia), and nano-silica (NS, Zhengzhou Dongshen Petrochemical Technology, China). The OPC (Type 1) and class F FA conform to the requirements of ASTM C150 [26] and ASTM C618 [27], respectively; the chemical and physical properties are shown in Table 1. The properties of the nano-silica are shown in Table 2. River sand (Tronoh, Perak, Malaysia) with a specific gravity of 2.65 g/cm^3^, fineness modulus of 2.86, and water absorption of 1.24% was utilized as a fine aggregate in accordance with the requirements of ASTM C33. Untreated Mesh 30 crumb rubber (CR, Heap Hoe Tyres Sdn Bhd, Kedah, Malaysia) with a specific gravity of 0.95 g/cm^3^ was used as a partial replacement to sand by volume. The gradation curves for fine aggregates and crumb rubber are shown in Figure 1, which were determined in accordance with the requirements of ASTM C136/C136M-14 [28].

PVA fibers (12 mm long and 40 μm circular cross-sectional diameter, (Kuraray, Okayama, Japan) were used as the primary reinforcement for the rubberized ECC mixture; the physical properties of the PVA fibers are shown in Table 3. Polycarboxylate based superplasticizers (Sika Viscocrete-2044, Sika Kimia Sdn Bhd, Negeri Sembilan, Malaysia) with a pH value of 6.2, specific gravity of 1.08 g/cm^3^, an absence of chloride ion content, and a density of 1.11 kg/L were used to achieve the desired flowability for all mixtures, as the water content was kept constant. Based on trial and errors mixtures, the water-cement ratio was limited to 0.15 in the rubberized ECC mixture to avoid bleeding and segregation, as well as to ensure paste workability to achieve the target compressive strength of 73.5 MPa.

### 2.2. Rubberized ECC Mixtures Proportions Using RSM

Response surface methodology (RSM) is the most adequate computational and mathematical approach used and is most widely used for the analysis and design of models that relate and respond to one or more independent factors. RSM also used to optimize models with multiple objectives by specifying desirable objectives according to responses or factors [29,30]. Various design forms, including central composite, Box-Behnken, and historical data, are usable for RSM analyses, which might be utilized to establish statistical relationships between responses and independent factors. The selection of a design model is based on several responses and factors [30].

In this study, Design Expert software (Design Expert v10, StatEase, Godward, MN, USA) was used for RSM optimization and the mixtures proportions. The central composite design (CCD) approach was used for designing the experiments based on four factors, namely CR, fly ash, PVA, and NS. Three variations of CR were used as partial replacements to fine aggregates (0, 2.5, and 5% by volume) and fly ash was used as a partial replacement to cement (OPC) (0, 25 and 50% by weight). As for the addition of nano-silica and PVA fiber, the variations were 0, 1, and 2% by weight of cementitious material. A total of 30 trial mix designs, along with their proportions, were then produced using RSM. For each mix, the water-cement ratio was kept constant at a value of 0.15. For each mix, the flexural strength, direct tensile strength, elastic modulus, Poisson’s ratio, creep, and drying shrinkage were tested in a lab, and were considered as the responses for RSM analyses and mixture optimizations. The best mix design and optimization model were then determined. The total mixture constituents are depicted in Table 4.

### 2.3. Specimens Preparation and Test Procedures

Each mixture was prepared in the following order: the ingredients were first weighed with a weighing scale, then the dry materials of cement, fine aggregates, crumb rubber and fly ash were mixed under dry conditions using a pan type concrete mixer (UTEST, Ankara, Turkey) with continuous stirring for about 1–2 min. Water and superplasticizer were then slowly added and the mixing continued for another 5 min. At this stage, after one minute, the nano-silica and PVA were added. PVA fibers were added gradually to avoid balling effects and then mixing continued for another 2–3 min. After measuring the flowability of the mixture, the molds were prepared according to each testing standard and then kept in a curing room for 24 h. Samples were then demolded and kept in a curing water tank at 23 °C until a testing age of 28 days of curing.

Compressive strength tests were conducted in accordance with requirements of ASTM C109/C109M [31]. Three 50-mm cubic samples were tested at 28 days using a 3000kN capacity digital Universal Testing Machine (UTM, ELE, Leighton Buzzard, UK).

**Figure 2:**
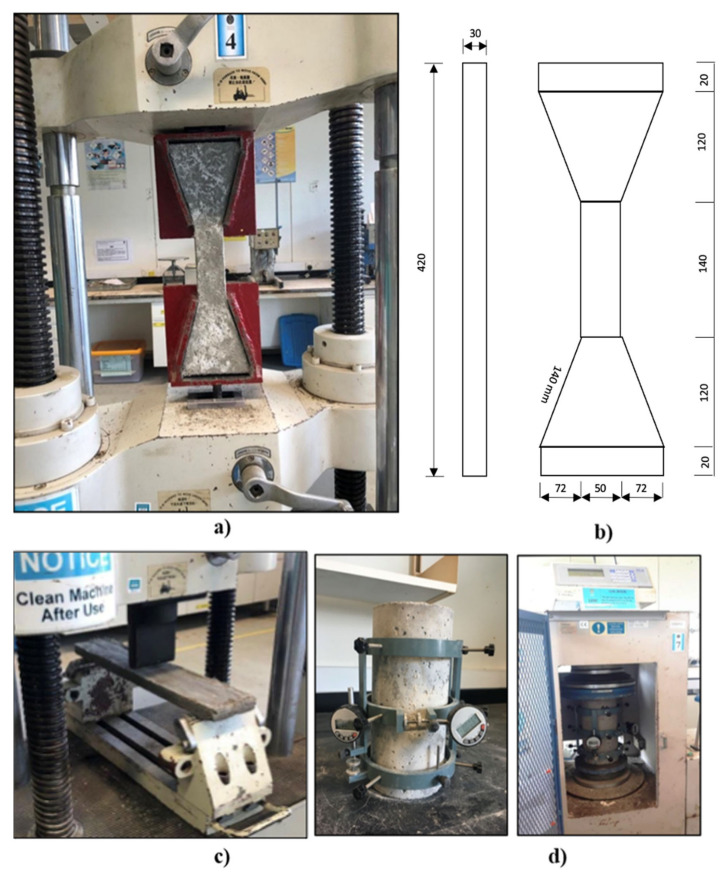
Experimental testing setup for the (**a**) direct tensile test (dog-boned); (**b**) dog-boned-shaped dimensions (unit = mm); (**c**) three-point bending test; and (**d**) elastic modulus and Poisson’s ratio.

The direct tensile strength was carried out on dog-boned-shaped samples using a uniaxial tensile testing machine (GOTECH, Taichung City, Taiwan), as shown in Figure 2a,b, in accordance with the requirements and recommendations of Rokugo [32]. A total of 3 specimens for each mix was prepared and tested at 28 days of curing. The gauge length of the specimens was 80 mm and loading were applied at a controlled displacement rate of 0.005 mm/s. The load and displacement values were measured using a longitudinal potential displacement transducer (LPDT, GOTECH, Taichung City, Taiwan) that was built into the machine and the measurements were captured using a computer data recording system (U60 v6.1 Software, Gotech Testing Machines Inc, Taichung City, Taiwan).

For the flexural strength test, a 200kN capacity digital Universal Testing Machine (UTM, GOTECH, Taichung City, Taiwan), three beams per mix, with dimensions of 500 mm × 100 mm × 25 mm, as shown in Figure 2c, were prepared and tested under a three-point bending test in accordance with the requirements of ASTM C293/C293M [33] at a uniform rate of stress 0.06 MPa/s.

The elastic modulus and Poisson’s ratio of rubberized ECC were measured using testing cylinder samples with a 150 mm diameter and 300 mm height at 28 days in accordance with the requirements of ASTM C469/C469M-14 [34] (as shown in Figure 2d). The 3000kN capacity digital Universal Testing Machine (UTM, ELE, Leighton Buzzard, UK) was fitted with a longitudinal compressometer to measure the vertical strain and an extensometer to measure the lateral strain. To measure the Poisson’s ratio, a load was applied to the specimens at a rate of 35 ± 5/s psi. The applied load and its corresponding deformation were recorded. In addition, when the applied load has reached 40% of the ultimate load, the elastic modulus can then be calculated.

**Figure 3:**
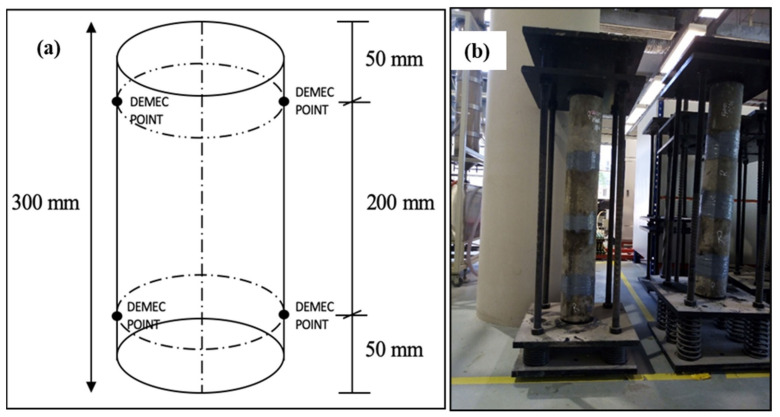
(**a**) Demountable mechanical strain gauge point positions on creep specimen and (**b**) creep loading frame.

For the creep test, once the optimized mix proportions were obtained, six cylindrical specimens (150 mm × 300 mm) were prepared in accordance with the requirements of ASTM C512/C512M-15 [35]. Two samples were used for compressive strength and the remaining four samples were used for the creep test. After casting, the specimens were stored in a curing room at a temperature of approximately 23 °C for 24 h, then demolded and cured in a clean water tank at 23 °C for 7 days. Four demountable mechanical (DEMEC) gauge points were attached, i.e., two on each diametrically opposing side at 200 mm, as shown in Figure 3a. Two spring loaded loading frames (Wuxi, China) were used and comprised four vertical threaded shafts. Four springs are attached at the lower ends of the shafts, sandwiched between two bearing plates (upper base plate and lower base plate), as shown in Figure 3b. The springs were connected for the application and maintained the applied loads within a scope of ±2% in the event of any change in the dimensions of the specimens. Loading was applied by means of a hydraulic pump, two jack plates (upper and lower), which were spaced at a distance that could be adjusted depending on the size of the hydraulic jack pump. To prevent eccentricity in the samples, two loading plates were attached to ensure the specimens could be positioned on the loading frame without much movement. The distance between loading frames did not exceed 1780 mm. Finally, to achieve a smooth surface for the specimens and to promote uniform distribution of stresses, the bottom and top of each sample were capped using a two-part epoxy resin, Sikadur -330 where it was prepared by mixing (component A) with a hardener (component B) at a weight ratio of 4:1. For each mix, the specimens were connected (top to bottom) with the epoxy resin before the plugs were placed. The whole assembly was then placed and aligned into the creep loading frame, as shown in Figure 3b. Creep was calculated using Equation (1), and the coefficient of creep was calculated using Equation (2).

(1) $$C\left(t_{k},t_{0}\right)=\varepsilon_{t}\left(t_{k}\right)\;–\;\varepsilon_{ie}\left(t_{0}\right)\;–\;\varepsilon_{sh}\left(t_{k}\right)$$

(2) $$\phi \left(t_{k},\;t_{0}\right)=\frac{C\left(t_{k},t_{0}\right)}{\varepsilon_{ie}\left(t_{0}\right)}$$

where $C\left(t_{k},t_{0}\right)\;$is total creep at time $t_{k}$ due to the applied stress at time $t_{0}$; $\varepsilon_{t}\left(t_{k}\right)$ is total strain at time $t_{k}$; $\varepsilon_{ie}\left(t_{0}\right)\;$ is initial instantaneous elastic strain at time $t_{0}$; $\varepsilon_{sh}\left(t_{k}\right)$ is corresponding shrinkage strain for the same specimen at time $t_{k}$; and $\phi \left(t_{k},\;t_{0}\right)\;$is creep coefficient at any time, $t_{k}$.

Finally, the drying shrinkage was carried out with prisms with dimensions of 75 mm × 75 mm × 300 mm. Shrinkage deformation was determined by the change in length of the specimens (difference in initial length and final length after air-drying age of 28 days). A total of three specimens for each mixture were tested with accordance with ASTM C490/C490M-17 [36].

## 3. Results and Discussions

### 3.1. Creep Test Results

**Figure 4:**
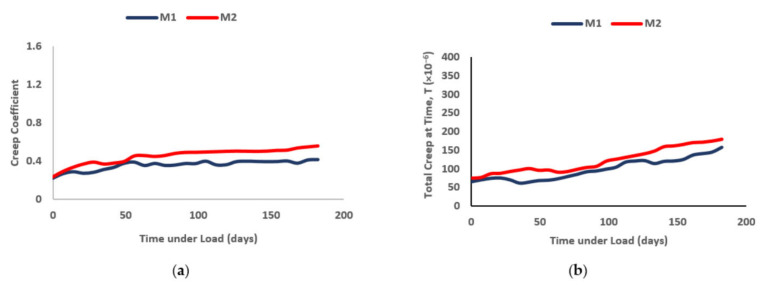
(**a**) Creep strain and (**b**) creep coefficient of rubberized ECC containing nano-silica.

The creep measurement for the ECC mixtures were reported in two forms, total creep strain and creep coefficients, as shown in Figure 4. Both ECC mixtures show an increase in creep strain and creep coefficient over time. The rate of increase was higher in the early stages and gradually followed a steady incline throughout the rest of the testing period. This was bound to happen, as during the early stages of concrete, the compressive strength of concrete is very low, and thus, deformation could occur a great deal easier when loading is applied. In addition, as time continues, the hydration of cement takes place, which, in turn, results in the ECC gaining strength, which also, in turn, increases the elastic modulus and consequently reduces deformation under the long duration of applied loading. The addition of nano-silica decreased the overall creep strain and creep coefficient for both ECC mixtures. When comparisons were made, the total creep of M2 to M1 showed higher percentages, by 17.64, 68.6, 22.24% at the 7th, 30th and 90th days, respectively. The creep coefficient, on the other hand, for M1, ranged from 0.225 to 0.415, whereas, for M2, it ranged from 0.234 to 0.555. It is noticeable that there was a slight difference between the creep coefficient ranges between M1 and M2, and this was attributed to the fact that, when crumb rubber was added (lower stiffness compared to fine aggregates), it lowered the overall creep of the ECC. M1 and M2 had equal amounts of crumb rubber added into the mixture, hence, justifying the similarities in terms of ranges for creep coefficients of M1 and M2.

### 3.2. Response Surface Methodology (RSM)

RSM is the most adequate computational and mathematical approach used and the most widely used for analyses and the design of models that relate and respond to one or more independent factors [37]. The analysis of RSM involves developing a sequence of tests and gathering the experimental outcomes as answers. The operation is launched using response surface modeling, in which the central composite design (CCD) model was selected to suit the information set. For each variable, three levels of study were considered, at low, medium, and high levels. This was to provide an overall response at only one center point. The experimental design matrix and the experimental results are shown in Table 5.

### 3.3. Analysis of Variance (ANOVA)

Table 6 shows that the analysis of variance (ANOVA) results for the quadratic response models demonstrate significant ability of the developed models in estimating the properties of rubberized ECC containing nano-silica. The models’ F-values of 16.36, 3.15, 16.31, 5.28, 61.52, and 3.15, for compressive strength, elastic modulus, Poisson’s ratio, direct tensile strength, drying shrinkage, and flexural strength, respectively, indicated that the models were all significant, with only a 0.01% probability value (*p*-value) for all models. The significance of all factors, including models and their terms, could be examined using a 95% of confidence level (CI), where the *p*-value should be less than 0.05. For compressive strength, the model and the terms *A*, *B*, *C*, *AB*, *BD*, *B*^2^, and *D*^2^ were all significant as their *p*-values were less than 0.05, whereas, the *p*-values for the terms *D, AC, AD*, *BC*, *CD*, *A*^2^, and *C*^2^ were greater than 0.05, which means they are all not significant. For elastic modulus, the model and its terms *A, B*^2^, *C*^2^, and *D*^2^ were significant because they had *p*-value <0.05, but the rest of the model terms (*B*, *C*, *D*, *A*^2^, *AB*, *AC*, *AD*, *BC*, *BD*, and *CD*) were insignificant due to the *p*-value being >0.05. For the Poisson’s ratio, the model and terms *A, C, D, C^2^, AD, BD*, and *CD* were significant, while the model terms *B*, *A*^2^, *B*^2^, *C*^2^, *D*^2^, *AB*, *AC*, and *BC* were not significant with *p*-values of >0.05. For the direct tensile strength, the model and terms *C*, *D*, *AD*, *BD*, and *D*^2^ had *p*-values less than 0.05, which were significant, while the remaining models’ terms were insignificant. For the drying shrinkage model, only terms *A*, *C*, *D*, *AC*, *CD*, *A*^2^, and *D*^2^ were significant with *p*-values <0.05, whereas the rest of its terms were insignificant, with *p*-values >0.05. Finally, only four models’ terms of flexural strength *A*, *B*^2^, *C*^2^, and *D*^2^ were significant while the remaining ten terms were not significant with *p*-values >0.05. Based on ANOVA analysis, the proposed models that were created using Design Expert software assumed quadratic models and the empirical interactions between each input factor and output factor are as shown in Equations (3)–(8) (for compressive strength (*CS*), elastic modulus (*EM*), Poisson’s ratio (v), direct tensile strength (*DT*), drying shrinkage (*DS*), and flexural strength (*FS*), respectively). The positive and negative symbols before the model term represent antagonistic and synergistic effects of the independent factors on the responses of rubberized ECC.

(3) $$CS\;\left(MPa\right)=65.14-15.56A-3.29B-2.77C+0.62D+3.89A*B+0.76A*C-0.84A*D+1.97B*C+4.59B*D+0.626C*D+0.045A^{2}-7.66B^{2}-4.74C^{2}+14.76D^{2}$$

(4) $$EM\;\left(MPa\right)=25.08-2.13A+0.61B+0.45C-0.104D-0.59A*B-0.03A*C-0.51A*D-0.32B*C-0.46B*D-0.43C*D-0.019A^{2}-4.66B^{2}+4.33C^{2}-3.18D^{2}$$

(5) $$v=0.25-0.029A-0.0016B-0.01C-0.013D+0.0016A*B-0.0044A*C+0.027A*D+0.0045B*C-0.0079B*D-0.011C*D-0.0097A^{2}+0.01B^{2}+0.02C^{2}-0.0062D^{2}$$

(6) $$DT\;\left(MPa\right)=2.74-0.019A-0.021B+0.13C+0.15D-0.076A*B+0.014A*C+0.266A*D-0.0099B*C+0.15B*D+0.01C*D-0.063A^{2}+0.076B^{2}+0.071C^{2}+0.337D^{2}$$

(7) $$\mathrm{DS}\;\left(µE\right)=1274+110.1A+6.68B-8.3C+8.9D-2.66A*B+13.14A*C-0.102A*D+3.01B*C-2.01B*D+13.41C*D-64.6A^{2}+4.37B^{2}-1.1C^{2}+89.73D^{2}$$

(8) $$FS\;\left(MPa\right)=10.5+0.54A+0.041B-0.027C+0.524D-0.038A*B-0.048A*C-0.138A*D-0.231B*C-0.133B*D-0.03C*D-1.31A^{2}+0.113B^{2}-0.011C^{2}+0.803D^{2}$$

The developed models presented in Equations (3)–(8) showed insignificant terms (*p*-value > 0.05) which meant that these terms should be omitted from the equations. Therefore, the final models after removing all insignificant terms are presented as Equations (9)–(14), for compressive strength (*CS*), elastic modulus (*EM*), Poisson’s ratio (v), direct tensile strength (*DT*), drying shrinkage (*DS*), and flexural strength (*FS*), respectively.

(9) $$CS\;\left(MPa\right)=65.14-15.56A-3.29B-2.77C+3.89A*B+4.59B*D-7.66B^{2}+14.76D^{2}$$

(10) $$EM\;\left(MPa\right)=25.08-2.13A-4.66B^{2}+4.33C^{2}-3.18D^{2}$$

(11) v=0.25−0.029A−0.01C−0.013D+0.027A*D−0.0079B*D−0.011C*D

(12) $$DT\;\left(MPa\right)=2.74+0.13C+0.15D+0.266A*D+0.15B*D+0.337D^{2}$$

(13) $$\mathrm{DS}\;\left(µE\right)=1274+110.1A-8.3C+8.9D+13.14A*C+13.41C*D-64.6A^{2}+89.73D^{2}$$

(14) $$FS\;\left(MPa\right)=10.46+0.54A+0.11B^{2}-0.011C^{2}+0.803D^{2}$$

To confirm the adequacy, fitness, and consistency of these models, a degree of determination (correlation) was used. Table 6 shows a summary of the response model validation. The R^2^ values were significant for all models, which were greater than 90% (R^2^ > 0.9). The high values of the determination coefficient of R^2^ symbolized a good agreement between the determined responses to the proposed models in estimating the properties of rubberized ECC containing nano-silica. Consequently, all models were statistically satisfactory. Adequate precision (AP) was also determined to measure the ratio of signal to noise where every response was more significant than the desired value, as the AP value for all models was greater than 4. Furthermore, all models’ variabilities were also tested using their standard deviations (S.D.) and coefficients of variation (C.V.) with reference to the experimental results. In addition to the mean (μ) of the models, the low S.D. values revealed that the experimental results were more correlated to the developed models, which mean both results fit. Therefore, it could be concluded that the developed models can be employed to command the design space as seen in Table 7.

**Figure 5:**
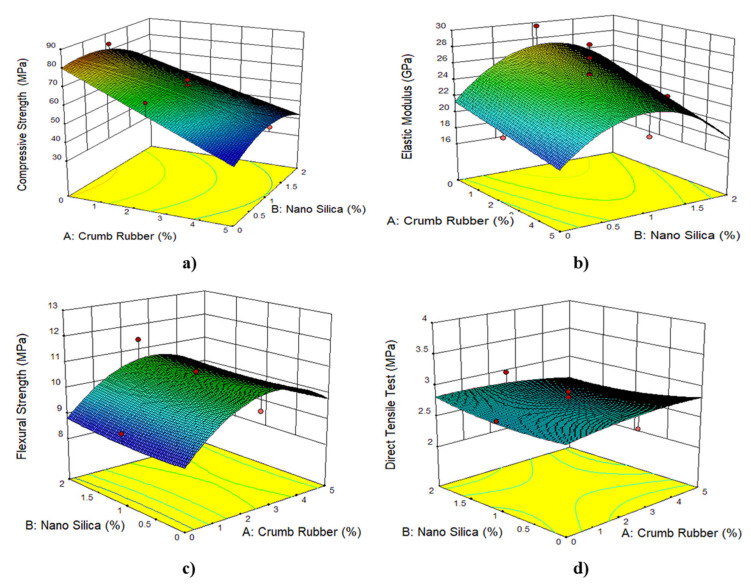
The 3D response surface plots for 50% fly ash and 1% PVA fiber for (**a**) compressive strength, (**b**) flexural strength, (**c**) direct tensile strength, and (**d**) elastic modulus.

**Figure 6:**
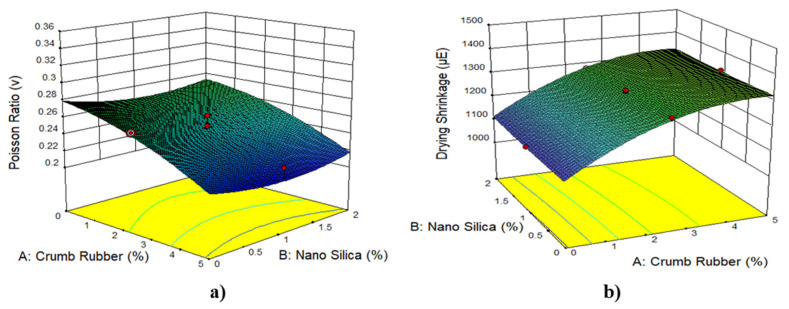
The 3D response surface plots for 25% fly ash and 1% PVA fiber for (**a**) Poisson’s ratio and (**b**) drying shrinkage.

**Figure 7:**
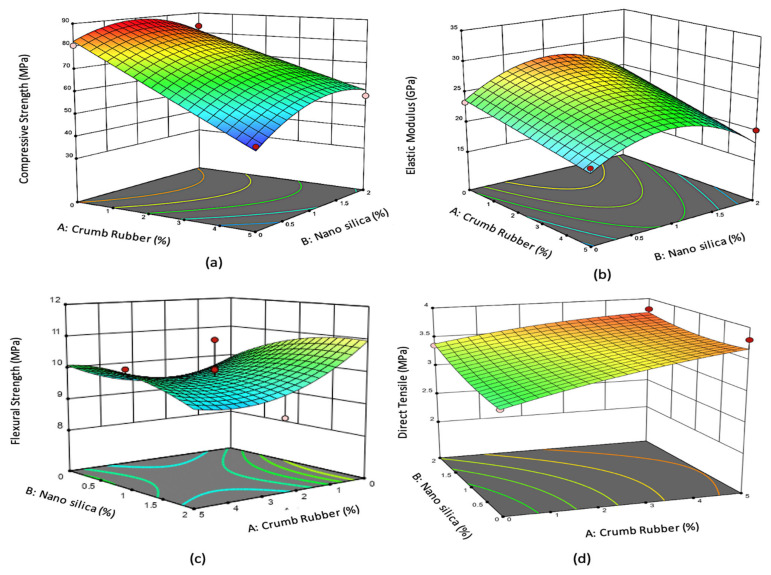
The 3D response surface plot for 50% fly ash and 2% PVA fiber for (**a**) compressive strength, (**b**) flexural strength, (**c**) direct tensile strength, and (**d**) elastic modulus.

**Figure 8:**
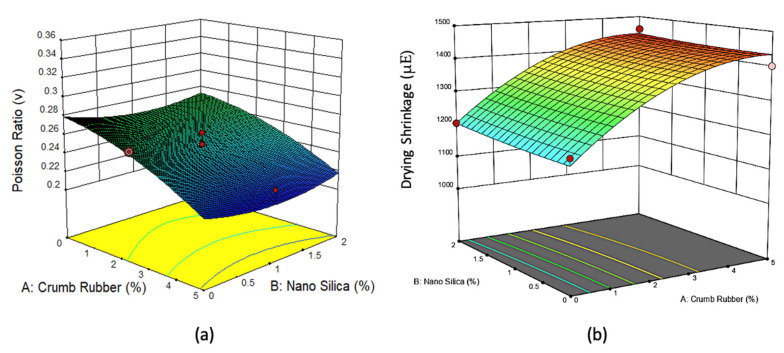
The 3D response surface plot for 50% fly ash and 2% PVA fiber for (**a**) Poisson’s ratio and (**b**) drying shrinkage.

Figure 5, Figure 6, Figure 7 and Figure 8 show the 3D response surface plots for compressive strength, flexural strength, direct tensile strength, elastic modulus, Poisson’s ratio, and drying shrinkage of rubberized ECC. As shown in Figure 5, the compressive strength, flexural strength, direct tensile strength, and elastic modulus of the rubberized ECC decreases with increasing crumb rubber content. This reduction is attributed to the hydrophobic nature of crumb rubber, which repels water and entraps air on its surface leading to increasing air voids inside the hardened ECC cement matrix, which thickens and weaken the interfacial transition zone (ITZ) between the CR particles and the hardened cement matrix. However, adding nano-silica to the mixture leads to an increase in compressive strength, flexural strength, direct tensile strength, and elastic modulus of the rubberized ECC due to the pozzolanic reaction of nano-silica with surplus Ca(OH)_2_ from cement hydration and produces more calcium-silicate-hydrate (C–S–H) gel, resulting in increased strength through densification of the microstructure of the hardened cement matrix and also the ITZ. On the other hand, Figure 6 shows that the Poisson’s ratio decreases, and the drying shrinkage increases with increasing CR content. It also shows that the addition of nano-silica increases the Poisson’s ratio and decreases the drying shrinkage due to the reduction of the permeability because of the formation of secondary C–S–H from the high pozzolanic reaction of nano-silica. The formed C–S–H gel fills up the capillary pores in the rubberized ECC matrix and thus reduces drying shrinkage.

### 3.4. Multi-Objective Optimization

The optimization process aimed to identify the optimum values (factors proportion) using the response surface of the chosen model to accomplish an optimized rubberized ECC mix. To acquire all the different combinations of outcomes, all factors were defined in the optimization criteria and a target compressive strength of 50 MPa was chosen; the responses were either maximized or minimized, as shown in Table 8. The creep test response was not included in the multi-objective optimization as it was conducted after the optimized rubberized ECC mixtures were obtained. The resulting optimized mix proportions were then used to conduct the creep test. By utilizing RSM using the Design Expert software, the optimization process and the desirability function were carried out. To validate each optimized rubberized ECC mixture, as well as to obtain a consistent result that was not altered by random events, the first three outcomes with the highest desirability value of 1 were chosen.

Table 9 presents the selected multi-objective optimization results of the rubberized ECC mixtures that gave a desirability of 1. The optimized mixture of rubberized ECC containing nano-silica was accomplished via partial substitution of fine aggregates with crumb rubber at 3.4% by volume and replacing 42.14% of the cement with fly ash by volume, as well as the addition of 1.345% and 1.245% of nano-silica and PVA fibers by weight of cementitious materials, respectively. The optimized outcome had a desirability of 1.0%. The corresponding results of the optimized mix for all the responses are shown in Table 9.

### 3.5. Model Validation

The optimized results and all model responses were then validated via experimental works, and the average results were compared to determine the percentage error for both experimental and optimized values (predicted). Table 10 shows the percentage difference between the experimental and predicted model results. The maximum percentage error across every response was 9.38%, which validated a good correspondence of the experimental, as well as the optimized results. This indicated that the proposed model for this research using RSM is highly reliable and can be used to predict the properties of rubberized ECC containing nano-silica.

## 4. Conclusions

Based on the results of the experimental and statistical analyses, the following conclusions can be drawn:

1. Incorporation of nano-silica can offset the adverse effects of crumb rubber content on the properties of rubberized ECC.
2. The reduction in the elastic modulus of rubberized ECC is attributed to the elastic properties of the crumb rubber particles, which act as tiny springs inside the hardened cement matrix. However, this adverse effect has been offset by adding nano-silica which restricted the internal structure of the rubberized ECC.
3. The developed quadratic equations can be used to predict the responses. All the responses are within the 5% significance level, where the probability value (*p*-value) is less than 0.05, clearly indicating that the models are significant with an outstanding 95% confidence level. The developed models using the RSM can provide reliable and accurate responses and thus can be used to predict the strength of the rubberized ECC containing NS.
4. The optimum mixture of rubberized ECC mixture achieved using RSM is highly reliable.

## Figures and Tables

**Table 1:** Chemical composition of cementitious materials.

Configuration	Cement (%)	Fly Ash (%)
SiO_2_ (%)	25.21	58.35
Al_2_O_3_ (%)	4.59	20.96
Fe_2_O_3_ (%)	2.99	4.9
CaO (%)	62.85	9.79
MgO (%)	1.70	1.99
Na_2_O (%)	0.98	2.41
K_2_O (%)	1.68	1.60
Specific gravity (g/cm^3^)	3.15	2.38
Loss on ignition (%)	2.2	1.25

**Table 2:** Nano-silica properties.

Item	Unit	Quality
Appearance	-	High-dispersive white powder
Hear reduction	%	≤3
Ignition loss	%	≤6
SiO_2_	%	≥99.8
Specific surface area	m^2^/g	100 ± 25
pH value	-	6.5–7.5
Surface density	g/mL	≤0.15
Dispensability (CCl_4_)	%	≥80
Oil-absorbed value	mL/100 g	≥250
Average particle size	nm	10−25
Hydrophobicity	-	Strong

**Table 3:** PVA fiber physical properties.

Fiber Type	Specific Gravity (g/cm^3^)	Elastic Modulus (GPa)	Length (mm)	Diameter (μm)	Aspect Ratio (L/d)	Tensile Strength (MPa)
PVA Fiber	1.3	41	12	40	462	1600

**Table 4:** Mixture constituents of each rubberized ECC model.

Mix		Factors (%)				Quantities (kg/m^3^)						
	w/cm	CR	FA	PVA	NS	OPC	FA	Fine Aggregate	CR	PVA	NS	Water
M1	0.15	0	0	1	0	260.00	0.00	210.60	0.00	5.85	0.00	39.00
M2	0.15	2.5	50	1	1	130.00	130.00	205.34	1.89	5.85	2.60	39.00
M3	0.15	0	50	0	2	130.00	130.00	210.60	0.00	0.00	5.20	39.00
M4	0.15	5	25	1	1	195.00	65.00	200.07	3.75	5.85	2.60	39.00
M5	0.15	2.5	25	1	1	195.00	65.00	205.34	1.89	5.85	2.60	39.00
M6	0.15	0	0	2	2	260.00	0.00	210.60	0.00	11.70	5.20	39.00
M7	0.15	2.5	0	1	1	260.00	0.00	205.34	1.89	5.85	2.60	39.00
M8	0.15	0	50	2	2	130.00	130.00	210.60	0.00	11.70	5.20	39.00
M9	0.15	5	50	0	0	130.00	130.00	200.07	3.75	0.00	0.00	39.00
M10	0.15	0	25	1	1	195.00	65.00	210.60	0.00	5.85	2.60	39.00
M11	0.15	2.5	25	1	1	195.00	65.00	205.34	1.89	5.85	2.60	39.00
M12	0.15	0	50	0	0	130.00	130.00	210.60	0.00	0.00	0.00	39.00
M13	0.15	2.5	25	1	1	195.00	65.00	205.34	1.89	5.85	2.60	39.00
M14	0.15	0	0	2	0	260.00	0.00	210.60	0.00	11.70	0.00	39.00
M15	0.15	2.5	25	1	1	195.00	65.00	205.34	1.89	5.85	2.60	39.00
M16	0.15	5	50	2	0	130.00	130.00	200.07	3.75	11.70	0.00	39.00
M17	0.15	2.5	25	1	1	195.00	65.00	205.34	1.89	5.85	2.60	39.00
M18	0.15	2.5	25	1	0	195.00	65.00	205.34	1.89	5.85	0.00	39.00
M19	0.15	5	50	2	2	130.00	130.00	200.07	3.75	11.70	5.20	39.00
M20	0.15	2.5	25	1	1	195.00	65.00	205.34	1.89	5.85	2.60	39.00
M21	0.15	0	50	2	0	130.00	130.00	210.60	0.00	11.70	0.00	39.00
M22	0.15	2.5	25	2	1	195.00	65.00	205.34	1.89	11.70	2.60	39.00
M23	0.15	2.5	25	1	2	195.00	65.00	205.34	1.89	5.85	5.20	39.00
M24	0.15	5	0	0	0	260.00	0.00	200.07	3.75	0.00	0.00	39.00
M25	0.15	5	50	0	2	130.00	130.00	200.07	3.75	0.00	5.20	39.00
M26	0.15	5	0	0	2	260.00	0.00	200.07	3.75	0.00	5.20	39.00
M27	0.15	0	0	0	2	260.00	0.00	210.60	0.00	0.00	5.20	39.00
M28	0.15	5	0	2	2	260.00	0.00	200.07	3.75	11.70	5.20	39.00
M29	0.15	2.5	25	0	1	195.00	65.00	205.34	1.89	0.00	2.60	39.00
M30	0.15	5	0	2	0	260.00	0.00	200.07	3.75	11.70	0.00	39.00

w/cm: water-cementitious ratio, CR: crumb rubber, FA: fly ash, PVA: Polyvinyl-alcohol fiber, NS: nano silica, OPC: ordinary portland cement, FA: fly ash.

**Table 5:** Experimental design matrix and responses for rubberized ECC mixture.

Mix	Factors (%)				Responses					
	A: CR	B: NS	C: FA	D: PVA	CS (MPa)	ME (GPa)	v	DT (MPa)	DS (µE)	FS (MPa)
M1	0	0	0	1	87.17	21.92	0.31	2.63	1103.70	8.95
M2	2.5	1	50	1	57.11	29.65	0.26	2.82	1267.80	10.75
M3	0	2	50	0	69.91	27.14	0.34	3.13	1150.20	8.80
M4	5	1	25	1	48.17	20.17	0.22	2.58	1323.90	9.50
M5	2.5	1	25	1	64.29	25.26	0.26	2.70	1260.30	10.75
M6	0	2	0	2	82.44	24.75	0.24	3.40	1204.50	10.20
M7	2.5	1	0	1	65.05	28.78	0.27	2.76	1284.40	10.35
M8	0	2	50	2	83.42	23.19	0.24	3.36	1203.70	9.95
M9	5	0	50	0	52.44	21.67	0.21	2.91	1402.90	10.25
M10	0	1	25	1	83.57	29.57	0.25	2.73	1101.30	9.01
M11	2.5	1	25	1	65.22	27.21	0.24	2.91	1281.30	10.10
M12	0	0	50	0	83.80	21.78	0.33	3.74	1124.80	8.89
M13	2.5	1	25	1	60.45	22.90	0.24	2.82	1279.20	10.05
M14	0	0	0	2	86.81	26.35	0.29	2.59	1203.50	9.87
M15	2.5	1	25	1	63.07	28.78	0.25	2.50	1283.30	10.15
M16	5	0	50	2	44.96	20.29	0.21	3.81	1401.30	11.71
M17	2.5	1	25	1	68.45	25.18	0.25	2.72	1280.10	10.25
M18	2.5	0	25	1	67.63	19.24	0.26	2.62	1281.30	9.95
M19	5	2	50	2	54.86	20.11	0.21	3.81	1459.20	10.05
M20	2.5	1	25	1	59.10	25.23	0.25	2.89	1254.10	10.25
M21	0	0	50	2	80.36	23.32	0.24	2.99	1203.50	10.20
M22	2.5	1	25	2	85.68	18.11	0.23	2.97	1361.10	12.20
M23	2.5	2	25	1	48.69	21.21	0.25	2.97	1281.90	11.40
M24	5	0	0	0	57.66	19.21	0.21	2.81	1399.80	9.95
M25	5	2	50	0	48.93	18.61	0.23	2.70	1403.30	10.05
M26	5	2	0	0	51.17	20.98	0.22	2.39	1402.40	10.70
M27	0	2	0	0	69.55	23.04	0.35	2.92	1250.30	8.90
M28	5	2	0	2	59.10	19.24	0.27	3.19	1401.90	11.35
M29	2.5	1	25	0	81.63	22.37	0.24	3.12	1358.80	11.10
M30	5	0	0	2	48.09	17.93	0.29	3.55	1424.30	10.990

A: crumb rubber, B: nano-silica, C: fly ash, D: PVA fiber, CS: compressive strength, ME: elastic modulus, v: Poisson’s ratio, DT: direct tensile strength, DS: drying shrinkage, and FS: flexural strength.

**Table 6:** ANOVA for the developed response models.

Response	Factors	Squares Sum	D.F.	Mean Square	F-Value	*p*-Value
Compressive strength	Model	5108.15	14	364.87	16.36	<0.0001
	*A*	4169.69	1	4169.69	186.94	<0.0001
	*B*	187.27	1	187.27	8.40	0.0110
	*C*	131.61	1	131.61	5.90	0.0282
	*D*	6.04	1	6.04	0.27	0.6105
	*AB*	230.35	1	230.35	10.33	0.0058
	*AC*	8.81	1	8.81	0.40	0.5391
	*AD*	9.81	1	9.81	0.44	0.5173
	*BC*	59.18	1	59.18	2.65	0.1241
	*BD*	294.85	1	294.85	13.22	0.0024
	*CD*	5.48	1	5.48	0.25	0.6274
	*A* ^2^	0.0055	1	0.0055	0.00025	0.9877
	*B* ^2^	156.03	1	156.03	7.00	0.0184
	*C* ^2^	59.79	1	59.79	2.68	0.1224
	*D* ^2^	655.05	1	655.05	29.37	<0.0001
	Lack of Fit	277.67	10	27.77	2.44	0.0984
Elastic modulus	Model	272.60	14	19.47	3.15	<0.0001
	*A*	78.20	1	78.20	12.65	0.0029
	*B*	6.47	1	6.47	1.05	0.3224
	*C*	3.43	1	3.43	0.56	0.4676
	*D*	0.18	1	0.18	0.028	0.8686
	*AB*	5.24	1	5.24	0.85	0.3718
	*AC*	0.017	1	0.017	0.0027	0.9592
	*AD*	3.65	1	3.65	0.59	0.4544
	*BC*	1.54	1	1.54	0.25	0.6250
	*BD*	2.94	1	2.94	0.47	0.5013
	*CD*	2.64	1	2.64	0.43	0.5234
	*A* ^2^	0.00096	1	0.00096	0.00016	0.9902
	*B* ^2^	57.78	1	57.78	9.35	0.0080
	*C* ^2^	49.70	1	49.70	8.04	0.0125
	*D* ^2^	30.42	1	30.42	4.92	0.0424
	Lack of Fit	72.46	10	7.25	1.79	0.2707
Poisson’s ratio	Model	0.037	14	0.002659	16.31	<0.0001
	*A*	0.015	1	0.015000	91.89	<0.0001
	*B*	0.000043	1	0.000043	0.26	0.6155
	*C*	0.002041	1	0.002041	12.52	0.0030
	*D*	0.002748	1	0.002748	16.86	0.0009
	*AB*	0.000039	1	0.000039	0.24	0.6332
	*AC*	0.000294	1	0.000294	1.80	0.1995
	*AD*	0.010	1	0.010000	63.48	<0.0001
	*BC*	0.000311	1	0.000311	1.91	0.1876
	*BD*	0.000867	1	0.000867	5.32	0.0358
	*CD*	0.001820	1	0.001820	11.17	0.0045
	*A* ^2^	0.000248	1	0.000248	1.52	0.2360
	*B* ^2^	0.000294	1	0.000294	1.81	0.1989
	*C* ^2^	0.001141	1	0.001141	7.00	0.0183
	*D* ^2^	0.000117	1	0.000117	0.72	0.4104
	Lack of Fit	0.002191	10	0.000219	4.31	0.1603
Direct tensile strength	*Model*	3.53	14	0.25	5.28	<0.0001
	*A*	0.0068	1	0.0068	0.14	0.7116
	*B*	0.0081	1	0.0081	0.17	0.6857
	*C*	0.3	1	0.3	6.32	0.0238
	*D*	0.36	1	0.36	7.44	0.0156
	*AB*	0.089	1	0.089	1.88	0.1910
	*AC*	0.0033	1	0.0033	0.070	0.7949
	*AD*	0.99	1	0.99	20.86	0.0004
	*BC*	0.0015	1	0.0015	0.031	0.8615
	*BD*	0.31	1	0.31	6.60	0.0214
	*CD*	0.0016	1	0.0016	0.034	0.8556
	*A* ^2^	0.011	1	0.011	0.23	0.6399
	*B* ^2^	0.015	1	0.015	0.32	0.5790
	*C* ^2^	0.013	1	0.013	0.28	0.6039
	*D* ^2^	0.34	1	0.34	7.18	0.0171
	Lack of Fit	0.60	10	0.060	2.56	0.1551
Drying shrinkage	Model	279.40	14	19,957.70	61.52	<0.0001
	*A*	208.50	1	208.50	642.82	<0.0001
	*B*	768.45	1	768.45	2.37	0.1446
	*C*	1195.33	1	1195.33	3.68	0.0741
	*D*	1271.57	1	1271.57	3.92	0.0664
	*AB*	107.75	1	107.75	0.33	0.5730
	*AC*	2626.96	1	2626.96	8.1	0.0123
	*AD*	0.15	1	0.15	0.000454	0.9833
	*BC*	138.08	1	138.08	0.43	0.5240
	*BD*	56.32	1	56.32	0.17	0.6828
	*CD*	2513.31	1	2513.31	7.75	0.0139
	*A* ^2^	11,086.53	1	11,086.53	34.17	<0.0001
	*B* ^2^	50.78	1	50.78	0.16	0.6979
	*C* ^2^	3.23	1	3.23	0.009950	0.9219
	*D* ^2^	24,209.25	1	24,209.25	74.62	<0.0001
	Lack of Fit	4084.39	10	408.44	2.61	0.1505
Flexural strength	Model	272.60	14	19.47	3.15	<0.0001
	*A*	78.20	1	78.20	12.65	0.0029
	*B*	6.47	1	6.47	1.05	0.3224
	*C*	3.43	1	3.43	0.56	0.4676
	*D*	0.18	1	0.18	0.028	0.8686
	*AB*	5.24	1	5.24	0.85	0.3718
	*AC*	0.017	1	0.017	0.002700	0.9592
	*AD*	3.65	1	3.65	0.59	0.4544
	*BC*	1.54	1	1.54	0.25	0.6250
	*BD*	2.94	1	2.94	0.47	0.5013
	*CD*	2.64	1	2.64	0.43	0.5234
	*A* ^2^	0.000964	1	0.000964	0.000156	0.9902
	*B* ^2^	57.78	1	57.78	9.35	0.0080
	*C* ^2^	49.70	1	49.70	8.04	0.0125
	*D* ^2^	30.42	1	30.42	4.92	0.0424
	Lack of Fit	72.46	10	7.25	1.79	0.2707

*A*: crumb rubber, *B*: nano-silica, *C*: fly ash, *D*: PVA fiber, *A*^2^, *B*^2^, *C*^2^, and *D*^2^: second order effect, *AB*, *AC*, *AD*, *BC*, *BD*, and *CD*: interaction effects, *D.F*: degree of freedom, F-value: Fisher-statistical test values, *p*-value: probability values.

**Table 7:** Model validation.

Model	Compressive Strength	Modulus Elasticity	Poisson’s Ratio	Direct Tensile	Dry Shrinkage	Flexural Strength
R^2^	0.939	0.946	0.938	0.931	0.983	0.944
Adj. R^2^	0.881	0.909	0.881	0.874	0.967	0.898
Pred R^2^	0.696	0.284	0.591	0.086	0.863	0.201
AP	14.62	6.73	15.93	9.11	27.11	10.44
S.D.	4.72	2.49	0.013	0.218	18.01	0.461
μ	65.96	23.11	0.255	2.97	1287.9	10.22
C.V.%	7.16	10.76	5.01	7.36	1.40	4.52

R^2^: correlation degree, Adj. R^2^: adjusted correlation degree, Pred R^2^: predicted correlation degree, AP: adequate precision, S.D.: standard deviation, μ: mean, and C.V.: coefficient of variation.

**Table 8:** Criteria for multi-objective optimization.

Factors and Responses	Notation	Target	Lower Limit	Upper Limit
Crumb rubber, %	A	In range	0	5
Nano-silica, %	B	In range	0	2
Fly ash, %	C	In range	0	50
PVA fiber, %	D	In range	0	2
Compressive strength, MPa	CS	Target	50	50
Flexural strength, MPa	FS	Maximize	8.8	12.2
Direct tensile strength, MPa	DT	Maximize	2.394	3.812
Elastic modulus, GPa	ME	Maximize	17.93	29.65
Poisson’s ratio	v	Minimize	0.2089	0.347
Drying shrinkage, E	DS	Minimize	1101.29	1459.24

**Table 9:** Optimized rubberized ECC mixtures.

CR, %	NS, %	FA, %	PVA Fiber, %	CS, MPa	FS, MPa	DT, MPa	ME, GPa	v	DS, μE	Desirability, %
3.49	1.345	42.14	1.245	50.00	10.601	2.941	22.062	0.240	1301.16	1.0
4.43	1.085	43.51	1.079	49.10	10.059	2.853	25.973	0.218	1323.86	1.0
4.70	0.118	47.03	1.975	48.80	11.352	3.574	19.91	0.222	1427.21	1.0

**Table 10:** Validation of the experimental and predicted model.

CR, %	NS, %	FA, %	PVA Fiber, %	Results & Error	CS, MPa	FS, MPa	DT, MPa	ME, GPa	v	DS, μE
3.49	1.345	42.14	1.245	Predicted	50.00	10.601	2.941	22.062	0.240	1301.16
				Experimental	47.2	10.38	2.665	20.38	0.231	1226.96
				Error, %	5.60	2.08	9.38	7.62	3.75	5.70
4.437	1.085	43.51	1.079	Predicted	49.1	10.059	2.853	25.973	0.218	1323.86
				Experimental	45.76	9.62	2.748	23.61	0.200	1209.64
				Error, %	6.8	4.36	3.68	9.09	8.25	8.627
4.703	0.118	47.03	1.975	Predicted	48.8	11.352	3.574	19.91	0.222	1427.21
				Experimental	46.22	10.91	3.341	19.37	0.204	1349.77
				Error, %	5.28	3.89	6.52	2.71	8.10	5.43

## Data Availability

The data used to support the findings of this study are included within the article.
